# Effect of Therapeutic Exercise on the Management of Hyperkyphosis in Adolescence and Young Adulthood: A Systematic Review

**DOI:** 10.1002/pri.70078

**Published:** 2025-06-06

**Authors:** Lorena Fernández Fernández, Clara Rodríguez‐Gude, Iris M. de Oliveira

**Affiliations:** ^1^ Departamento Bioloxía funcional e ciencias da saúde Universidade de Vigo Pontevedra Spain

**Keywords:** adolescent, exercise therapy, kyphosis, spinal curvatures, young adult

## Abstract

**Background and Purpose:**

Thoracic hyperkyphosis is characterised by an increase in the normal angle of the thoracic kyphosis. Several factors make adolescents and young adults a potential risk group for postural hyperkyphosis. Among the treatment options, therapeutic exercise allows patients to take an active role, providing a number of beneficial effects. The aim of this systematic review was to evaluate the scientific evidence on the efficacy of therapeutic exercise for hyperkyphosis in adolescents and young adults.

**Methods:**

A search in six databases (PubMed, Medline, PEDro, Scopus, SPORTDiscus and ENFISPO) was performed during January and February 2023, following the Preferred Reporting Items for Systematic Reviews and Meta‐Analysis (PRISMA).

**Results:**

Nine randomised clinical trials were analysed. Regarding methodological quality, four studies were of fair quality and five were of good quality. In all studies, a significant improvement in thoracic kyphosis angle was obtained. In addition, improvements were found in different aspects related to hyperkyphosis, such as lumbar lordosis angle, balance, quality of life and pain, among others.

**Conclusions:**

Therapeutic exercise appears to be effective in the management of hyperkyphosis in adolescents and young adults. However, many variables related to hyperkyphosis are not subject to primary evaluation and the quality of the evidence found is fair to good.

## 1 Introduction

Thoracic hyperkyphosis is characterised by an increase in the normal thoracic kyphosis angle (TKA) beyond the normal range of 20°–40° (Elpeze and Usgu 2022; Feng et al. 2018; Seidi et al. 2014; Yazici and Mohammadi 2017). A distinction is made between congenital kyphosis, postural kyphosis and Scheuermann's disease, the latter being the most common cause of thoracic and thoracolumbar spine hyperkyphosis during adolescence (Bezalel et al. 2019; Luhmann and Skaggs 2014). In addition, several factors make adolescents and young adults a potential risk group for postural hyperkyphosis (Feng et al. 2018; Özdemir Görgü and Algun 2022; Toprak Çelenay and Özer Kaya 2017). These factors include poor ergonomics at work and school, sedentary lifestyles, high external loads on the spine and emotional stress (Feng et al. 2018; Özdemir Görgü and Algun 2022; Toprak Çelenay and Özer Kaya 2017).

Despite the fact that hyperkyphosis negatively affects the physical and psychological health, balance and quality of life of many people, there is no standardised protocol for correcting increased TKA (Bansal et al. 2014; Özdemir Görgü and Algun 2022). It is therefore essential to specify precise prevention and treatment strategies (Calvo‐Muñoz et al. 2011, 2012; Toprak Çelenay and Özer Kaya 2017).

Methods such as surgery, bracing, taping, manual therapy and therapeutic exercise are most commonly used to treat thoracic hyperkyphosis (Jenkins et al. 2021; Kamali et al. 2016). Surgical options generally have good results in terms of kyphosis reduction, pain and short‐term physical function (Li 2021). However, compared to conservative treatment, they have a higher risk of complications (Luhmann and Skaggs 2014; Mehdikhani et al. 2016; Yaman and Dalbayrak 2014). Therefore, surgical treatment of hyperkyphosis should be reserved for cases with conservatively intractable pain, progressive hyperkyphosis and/or severe neurological compromise (Li 2021; Luhmann and Skaggs 2014; Weiss et al. 2009; Yaman and Dalbayrak 2014). Braces in spinal disorders can be stressful for patients. Most are uncomfortable to use, resulting in poor compliance (Fan et al. 2020; Mehdikhani et al. 2016; Weiss et al. 2009). Regarding taping, there is still insufficient evidence to support the effects of this technique in hyperkyphosis (Bulut et al. 2019). Manual therapy is claimed to reduce pain, improve circulation, correct spinal alignment, facilitate joint movement, stretch shortened muscles, and reduce adhesions (Bennell et al. 2010; Kamali et al. 2016). However, unlike other treatment options, therapeutic exercise allows patients to take an active role, providing beneficial effects that are not achieved with passive support alone (Bansal et al. 2014; Jenkins et al. 2021). Therapeutic exercise has been shown to be a form of prevention or treatment for diseases such as obesity, heart disease, stroke, type 2 diabetes mellitus and certain cancers (Barker and Eickmeyer 2020). In addition, it can be used for acute, sub‐acute and chronic back pain (Zhou et al. 2024). Therapeutic exercise methods include strengthening exercises, stretching exercises, core stabilisation exercises, balance exercises, Pilates and others (Zhou et al. 2024).

A personalised exercise protocol specifically aimed at improving spinal mobility, postural alignment, muscle imbalances, spinal extensor muscle strength and respiratory function can significantly improve hyperkyphosis and most of its consequences (Bansal et al. 2014; González‐Gálvez et al. 2019; Jenkins et al. 2021).

There are two published systematic reviews on the effectiveness of exercise in thoracic hyperkyphosis (Bansal et al. 2014; González‐Gálvez et al. 2019). Bansal et al. (2014) evaluated the effectiveness of exercise only in older adults but included non‐randomised study designs and were unable to perform a meta‐analysis or make clear recommendations. González‐Gálvez et al. (2019) reported that exercise was effective in reducing TKA, but did not assess the strength of the evidence or report separately for different age groups or duration of treatment.

In a systematic review and meta‐analysis by Jenkins et al. (2021), the effectiveness of treatments to reduce thoracic hyperkyphosis was investigated, with both short‐ and long‐term exercise programmes found to be effective. However, it covers all ages and different treatment modalities, reporting low evidence on the use of exercise in adolescents and young adults. In another systematic review in people over 45 years old, exercise interventions improved factors such as TKA and the back extensor muscle strength or endurance, among others (Ponzano et al. 2021).

Thus, the aim of this systematic review was to evaluate the scientific evidence on the efficacy of therapeutic exercise for hyperkyphosis in adolescents and young adults.

## 2 Methods

### 2.1 Sources of Information

During the months of January and February 2023, a literature search was carried out in the databases PubMed, Medline, PEDro, Scopus, SPORTDiscus and ENFISPO. This systematic review was carried out in accordance with the Preferred Reporting Items for Systematic Reviews and Meta‐Analysis (PRISMA) (Page et al. 2021).

To design the review, the PICO strategy (Santos et al. 2007) was used where: P (population) is adolescents or young adults with hyperkyphosis; I (intervention) is therapeutic exercise; C (control or comparison) participants with hyperkyphosis receiving another intervention or no intervention; and, finally, O (outcomes) is represented by different variables included in the studies, the main one being the thoracic kyphosis angle, but also balance, quality of life or the lumbar lordosis angle, among others, are studied.

### 2.2 Search Equations

The MeSH terms ‘kyphosis’ and ‘exercise therapy’ as well as the free terms ‘kyphosis’, ‘hyperkyphosis’ and ‘exercise therapy’ were used for the search strategy as shown in Table 1.

**TABLE 1:** Databases and search equations.

Databases	Search equations
Pubmed	1. (‘Kyphosis’[Mesh]) AND ‘Exercise therapy’[Mesh] 2. (‘Kyphosis’[Mesh] OR ‘Hyperkyphosis’) AND (‘Exercise therapy’[Mesh] OR ‘Exercise therapy’)
Medline	1. (MH ‘Kyphosis’) AND (MH ‘Exercise therapy’) 2. (MH ‘Kyphosis’ OR ‘Hyperkyphosis’) AND (MH ‘Exercise therapy’ OR ‘Exercise therapy’)
PEDro	1. ‘Kyphosis’ AND ‘Exercise therapy’ 2. ‘Kyphosis’ OR ‘Hyperkyphosis’ AND ‘Exercise therapy’ 3. Abstract & tittle: Kyphosis 4. Abstract & tittle: Hyperkyphosis
Scopus	1. ‘Kyphosis’ AND ‘Exercise therapy’ 2. (‘Kyphosis’ OR ‘Hyperkyphosis’) AND ‘Exercise therapy’
SPORTDiscus	1. (DE ‘KYPHOSIS’) AND (DE ‘EXERCISE’) 2. (DE ‘KYPHOSIS’ OR ‘Hyperkyphosis’) AND (DE ‘EXERCISE’)
ENFISPO	1. kyphosis AND exercise 2. (‘Kyphosis’ OR ‘Hyperkyphosis’) AND Exercise

Abbreviations: DE, descriptor; Mesh o MH, Medical Subjects Headings; PEDro, Physiotherapy Evidence Database.

In the PEDro database, the terms ‘kyphosis’ and ‘hyperkyphosis’ were also used separately in order to increase the number of results.

In addition, to find other studies of interest, the bibliographic references of the literature consulted were reviewed.

### 2.3 Selection Criteria

The following inclusion criteria were used in this systematic review: (a) randomised clinical trial; (b) participants were adolescents and/or young adults (10–24 years) with hyperkyphosis; (c) participants had to perform or receive some form of therapeutic exercise combined or not with other treatment approaches; (d) the study assessed variables to measure the effect obtained, such as thoracic kyphosis angle, pain, balance, quality of life, among others; and (e) articles from the last 10 years published in English or Spanish. Any other publications that did not meet the inclusion criteria or were not accessible in full text were excluded.

Inclusion and exclusion criteria were used to review the titles and abstracts of all articles and when there was insufficient information for inclusion or exclusion of the manuscript, the full text of the article was read. Two authors independently extracted the study data. Reasons for exclusion were recorded. In cases of disagreement, a third author was consulted to make a final decision.

### 2.4 Methodological Quality

The methodological quality of the articles analysed was assessed using the PEDro scale (Maher et al. 2003), which consists of 11 criteria that aim to assess internal validity (criteria 2–9) and adequate statistical information to make the results interpretable (criteria 10 and 11). In addition, it presents an extra criterion (criterion 1), which comes from the Delphi scale and refers to the internal validity of each study but is not included in the final score of the PEDro scale. According to the score obtained, the methodological quality of the studies can be classified as poor (< 4 points), fair (4–5 points), good (6–8 points), or excellent (9–10 points) methodological quality.

**FIGURE 1:**
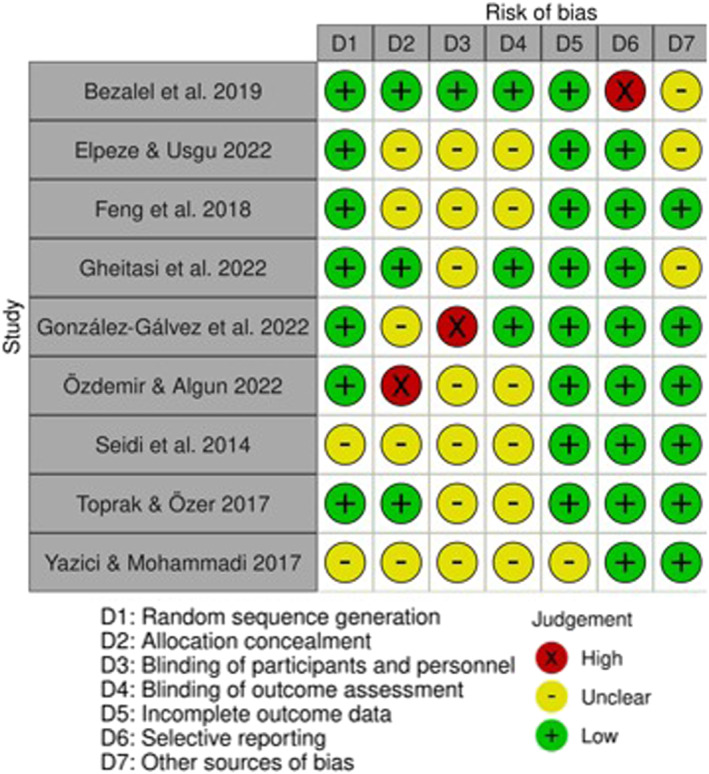
Cochrane risk of bias tool.

**FIGURE 2:**
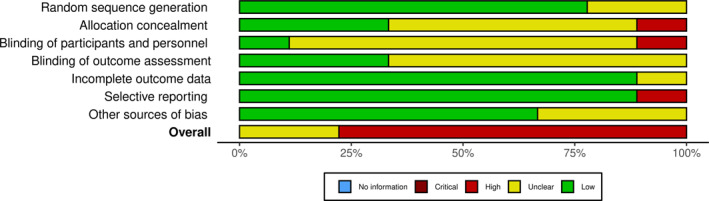
Cochrane risk of bias graph.

As a complement to this, an additional analysis was performed using the Cochrane Handbook (Higgins and Green 2011) for the analysis of the risk of bias, which can be illustrated in the traffic light in Figure 1 and the graph in Figure 2 made with the Risk‐of‐bias VISualization (robvis) tool (McGuinness and Higgins 2021).

## 3 Results/Findings

**FIGURE 3:**
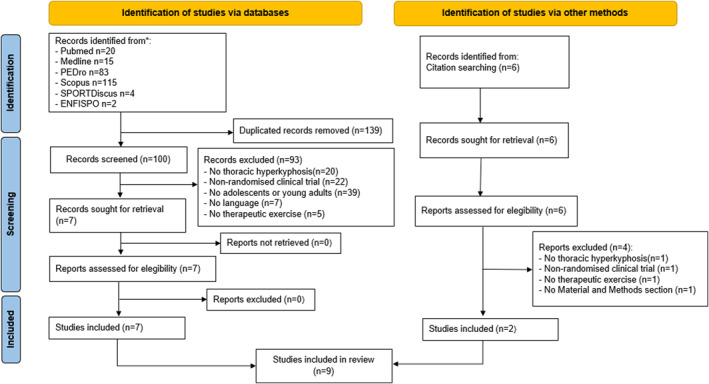
Flow diagram. *Filters used to obtain these results: Pubmed. 2012–2023; Article type: Randomised Controlled Trial; Age: Child: birth‐ 18 years, Young Adult: 19–24 years. Medline: Publication date: 2012–2022; Article type: Randomised Controlled Trial; Age: Child: birth‐ 18 years, Young Adult: 19–24 years. PEDro. Method: Clinical trial. Published since 2012. Scopus. 2012–2022. Keywords: Randomised Controlled Trial, adolescent, child, young adult. Sport Discus. Publication date: 2012–2022. Type: randomised controlled trials. ENFISPO. 2012–2023. Journal article.

The initial search using the equations performed in the different databases (Table 1) yielded 239 records. Of these, 139 were eliminated as duplicates. After applying the inclusion and exclusion criteria by title and abstract to the remaining 100 articles, 93 records were eliminated, leaving 7 articles to be read in full text and which were included because they met the criteria. In addition, 6 articles were identified from the literature review, of which 2 met the inclusion criteria after full text analysis. Therefore, 9 articles were finally included in this systematic review (Bezalel et al. 2019; Elpeze and Usgu 2022; Feng et al. 2018; Gheitasi et al. 2023; González‐Gálvez et al. 2022; Özdemir Görgü and Algun 2022; Seidi et al. 2014; Toprak Çelenay and Özer Kaya 2017; Yazici and Mohammadi 2017). Figure 3 shows the flow chart of the process.

The analysis of methodological quality, carried out with the PEDro scale, shows good quality (6–8 points) in five articles (Bezalel et al. 2019; Feng et al. 2018; Gheitasi et al. 2023; González‐Gálvez et al. 2022; Toprak Çelenay and Özer Kaya 2017) and fair quality (4–5 points) in four studies (Elpeze and Usgu 2022; Özdemir Görgü and Algun 2022; Seidi et al. 2014; Yazici and Mohammadi 2017) (Table 2).

**TABLE 2:** Results obtained after the evaluation of methodological quality using the PEDro scale.

PEDro scale												
Author	1	2	3	4	5	6	7	8	9	10	11	Total
Bezalel et al. 2019	1	1	1	0	1	0	1	1	1	1	1	8/10
Elpeze and Usgu 2022	1	1	0	1	0	0	0	0	0	1	1	4/10
Feng et al. 2018	1	1	0	1	0	0	0	1	1	1	1	6/10
Gheitasi et al. 2023	1	1	1	1	0	0	1	1	1	1	1	8/10
González‐Gálvez et al. 2022	1	1	0	1	0	0	1	1	1	1	1	7/10
Özdemir Görgü and Algun 2022	1	1	1	1	0	0	0	0	0	1	1	5/10
Seidi et al. 2014	0	1	0	1	0	0	0	1	0	1	1	5/10
Toprak Çelenay and Özer Kaya 2017	1	1	1	1	0	0	1	1	0	1	1	7/10
Yazici and Mohammadi 2017	0	1	0	1	0	0	0	0	0	1	1	4/10

*Note:* 1: Criteria were specified; 2: Subjects were randomly assigned to groups (in a crossover study, subjects were randomised as they received treatments); 3: Assignment was concealed; 4: Groups were similar at baseline with respect to the most important prognostic indicators; 5: All subjects were blinded; 6: All therapists who administered therapy were blinded; 7: All assessors who measured at least one key outcome were blinded; 8: Measures of at least one of the key outcomes were obtained from more than 85% of subjects initially assigned to groups; 9: Results were presented for all subjects who received treatment or were assigned to the control group, or when this could not be, data for at least one key outcome were analysed by ‘intention‐to‐treat’; 10: Results of statistical comparisons between groups were reported for at least one key outcome; 11: The study provides point and variability measures for at least one key outcome.

With regard to the analysis of the risk of bias according to Cochrane handbook, the traffic light in Figure 1 and the graph in Figure 2 show that attrition bias is the lowest risk in general. This is followed by reporting bias, with only one article (Bezalel et al. 2019) at high risk, and selection bias (random sequence generation), with two articles (Elpeze and Usgu 2022; Yazici and Mohammadi 2017) whose risk of bias is unclear. In contrast, an unclear risk for the majority of studies in conduct (Elpeze and Usgu 2022; Feng et al. 2018; Gheitasi et al. 2023; Özdemir Görgü and Algun 2022; Seidi et al. 2014; Toprak Çelenay and Özer Kaya 2017; Yazici and Mohammadi 2017) and detection (Elpeze and Usgu 2022; Feng et al. 2018; Özdemir Görgü and Algun 2022; Seidi et al. 2014; Toprak Çelenay and Özer Kaya 2017; Yazici and Mohammadi 2017) bias should be highlighted. That is, with respect to blinding of participants and staff and of outcome assessors.

The characteristics of the studies in terms of sample, variables, assessment tools and timing of measurement and intervention are represented in Table 3. The number of participants ranged from 40 in the study with the fewest participants (Yazici and Mohammadi 2017) to 170 in the study with the highest participation (Gheitasi et al. 2023). Regarding sex, the percentage of males ranged from 10% (Özdemir Görgü and Algun 2022) to 100% (Elpeze and Usgu 2022; Yazici and Mohammadi 2017), with equal participation between males and females in three of the studies (Gheitasi et al. 2023; Seidi et al. 2014; Toprak Çelenay and Özer Kaya 2017).

**TABLE 3:** Study characteristics.

Author	Sample	Variables, assessment tools and time of measurement	Intervention
Bezalel et al. 2019	*n* = 44 EG: 63.6%♂ 14.5 ± 1.8 y CG: 20%♂ 13.4 ± 1.7 y	**TKA y L5‐KAL**: Lateral X‐rays of the entire spine in ST. **QoL**: SRS‐22. **SPBI**: 0–10 (satisfied to very dissatisfied with own body perception) **Pain**: NPRS (0–10). **Shoulder flexion** (SUP and ST) and **Kyphotic deformity**: Digital inclinometer. **Home exercise record**. 3 assessments: Baseline, at 6 and 12 months.	1x/wk for 12 months. **EG**: 5 Schroth ex. (Corrective therapeutic exercises, breathing techniques and re‐education of the neuromuscular system). **CG**: 5 classical antigravitational ex (ST) Both were asked to perform exercises daily during the study period.
Elpeze and Usgu 2022	*n* = 62♂ CCEP: 21♂ 14.7 ± 1.3 y TEP: 22♂ 13.5 ± 1.1 y CG: 19♂ 13.9 ± 1.9 y	**TKA**: Mobile phone inclinometer and flexicurve. **Balance**: Baropodometry and Romberg index. At baseline and week 12. **PPT**: Record of average number of times/week that participant corrected posture.	**Ex. 40–45 min 3x/wk during 12 weeks**. **CCEP:** Cervical flexion, neck extensor and pectoral stretching in ST and SUP, and postural awareness training. **TEP:** Pectoral stretching and thoracic self‐mobilisation in ST and SUP. **CG**: No ex.
Feng et al. 2018	*n* = 164 EG: 51♂ 14.2 ± 1.4 y, 39♀ 14.0 ± 1.5 y; CG: 53♂ 13.5 ± 1.5 y, 38♀ 13.6 ± 1.5 y	**Spinal column shape and mobility** (TKA, LLA, SA, INA y ROM thoracic, lumbar and sacral): Spinal mouse. Baseline and week 9.	**EG**: functional ex. To recover ROM and improve muscle strength and proprioception. 15/20 min 2 x/wk for 8 wk. **CG**: Push‐ups, 50 m run and squats.
Gheitasi et al. 2023	*n* = 170 EG1: 29♂ + 28♀ 14.1 ± 1.3 y EG2: 28♂ + 28♀. 14.0 ± 1.3 y CG: 28♂ + 29♀. 13.8 ± 1.6 y	**TKA**: Gold standard Cobb angle derived from lateral standing with radiographs. Baseline and week 24.	**EG1**: Milwaukee brace (8 h/day in 1st wk. Increase t of use by 2 h/day up to 22 h/day) + EC (proprioception, strengthening, stretching, maintaining optimal posture) 60 min 3x/week for 24 wk. **EG2**: Brace only. **CG**: 1 deferred intervention after 6 months waiting list.
González‐Gálvez et al. 2022	*n* = 97 EG: 12♂ + 37♀. 13.4 ± 1.2 y CG: 14♂ + 34♀. 13.6 ± 1.2 y	**Sagittal spinal curvature** (TKA, LLA and INA in relaxed ST and in sit‐and‐reach): Spinal mouse. **Hamstring extensibility**: P‐SLR, A‐ SLR and sit‐and‐reach. Baseline and week 38.	**EG**: Pilates. 3 phases. Strengthening, trunk stability, mobilisation, breathing cycle and stretching. 15 min 2 times/week for 38 weeks. **CG**: 2 regular PE sessions.
Özdemir Görgü and Algun 2022	*n* = 63 6♂ + 57♀ PCEG: 20.4 ± 1.4 y SBTEG: 20.1 ± 1.2 y GC: 20.1 ± 1.0 y	**TKA y LLA**: Spinal mouse. **Balance**: Biodex balance system. QoL: SRS‐22. Baseline and week 9.	Ex 60 min 2 x/wk for 8 weeks. **PCEG**: Strengthening, stretching and breathing. **SBTEG**: Spinal stretching, sagittal correction with proprioceptive stimuli and breathing correction. **CG**: Information.
Seidi et al. 2014	*n* = 56 LCEP: 10♂ + 9♀. CCEP: 9♂ + 9♀. GC: 9♂ + 10♀. 20.9 ± 1.7 y	**TKA**: Flexicurve. **Forward head and shoulder angles:** Lateral photographic method. Baseline and week 12.	Ex. 3 x/wk for 12 wks. Warm‐up + 5 prescribed exs. + cool‐down. **LCEP**: Stretching, self‐mobilisation and strengthening. **CCEP**: In all exercises, chin flexion, thoracic spine erection and scapulae adduction. **CG**: No ex.
Toprak Çelenay and Özer Kaya 2017	*n* = 53 EG: 13♂ + 15♀. 21.0 ± 1.3 y CG: 13♂ + 12♀ 20.4 ± 1.2 y	S**pinal curvature (TKA y LLA)**: Spinal mouse. **Pain**: VAS. **Postural balance: Biodex balance system. Core strength: McGill trunk muscle endurance tests. Baseline and week 8**.	**EG**: ex. (Warm up 10 min, stabilisation 25 min, cool down 10 min) 3 phases of progression: Static, dynamic and functional. 45 min 3x/wk for 8 wks. **CG**: No ex.
Yazici and Mohammadi 2017	*n* = 40♂ EG: 20♂ 16.8 ± 1.8 y CG: 20♂ 17.5 ± 2.1 y	S**pinal curvature** (**TKA** y LLA): Spinal mouse. Baseline and week 8.	**EG**: CE (warm up 10 min, stretch 10 min, strengthen 30 min, cool down 10 min) 60 min 3x/wk for 8 wks. **CG**: No ex.

Abbreviations: A‐SLR, active straight leg raise; CCEP, Comprehensive Corrective Exercise Programme; CG, control group; EG, experimental group; ex, exercise; INA, incline angle; L5‐KAL, L5 kyphosis apex line; LCEP, local corrective exercise programme; LLA, lumbar lordosis angle; min, minutes; NPRS, Numerical pain rating scale; PCEG, postural corrective exercise group; PPT, postural perception training; P‐SLR, passive straight leg raise; QoL, quality of life; ROM, range of motion; SA, sacral angle; SBTEG, Schroth‐based three‐dimensional exercise group; SPBI, self‐perceived body image; SRS‐22, Scoliosis Research Society‐22 Questionnaire; ST, standing position; SUP, supine position; TEP, thoracic exercise programme; TKA, thoracic kyphosis angle; VAS, visual analogic scale; wk, week; y, Years.

The duration of the intervention ranged from 8 weeks (Feng et al. 2018; Özdemir Görgü and Algun 2022; Toprak Çelenay and Özer Kaya 2017; Yazici and Mohammadi 2017) to 1 year (Bezalel et al. 2019).

All studies measured TKA and most of them also measured LLA (Feng et al. 2018; González‐Gálvez et al. 2022; Özdemir Görgü and Algun 2022; Toprak Çelenay and Özer Kaya 2017; Yazici and Mohammadi 2017).

All participants in the intervention group showed significant improvements over the control group in some of the variables, with the exception of the study by Yazici and Mohammadi (2017). Table 4 details the intra‐group and inter‐group results of each study.

**TABLE 4:** Intra‐group and inter‐group results.

Author	Intra‐group results	Inter‐group results
Bezalel et al. 2019	EG and CG: significant changes over time in shoulder flexion, kyphotic deformity, SRS‐22 and thoracic kyphosis. EG: Significant improvement in SPBI. CG: Significant improvement in pain in the last week.	Significantly greater improvement in thoracic kyphosis and kyphotic deformity in favour of EG.
Elpeze and Usgu 2022	CCEP and TEP: Highly significant reduction in TKA. CCEP: Only one with improvement in Romberg index compared within group (*p* < 0.001), CG: No significant change.	Greater reduction of TKA in CCEP (*p* < 0.020). PPT improved in CCEP compared to other groups (*p* < 0.001).
Feng et al. 2018	EG and CG: significant difference in TKA and INA pre‐ and post‐test. EG: Significant difference in thoracic ROM and change in SA.	Post‐test TKA and change in SA and thoracic ROM significantly different in EG versus CG (*p* < 0.05) in favour of EG.
Gheitasi et al. 2023	EG1 and EG2: Significant decrease in TKA (15.5° and 8.8° respectively). CG: Slight increase in TKA (1.2°)	Significant differences between EG1 versus CG in favour of EG1; EG2 versus CG in favour of EG2 and EG1 versus EG2 in favour of EG1.
González‐Gálvez et al. 2022	EG: Significant reduction in TKA and LLA in relaxed standing position. Significant improvement in P‐SLR and A‐SLR tests. EG and CG: significant increase in thoracic curvature and INA in sit‐and‐reach.	Final diagnosis of hyperkyphosis in 53% of EG and 77% of CG participants. Adjusted mean differences between EG and CG in INA −2.9 (*p* = 0.03) and in P‐SLR and A‐SLR tests in favour of EG.
Özdemir Görgü and Algun 2022	Statistically significant decrease in TKA in PCEG and SBTEG and in LLA in SBTEG. Equilibrium and QoL: In results before and after intragroup test, significant differences in PCEG and SBTEG. CG: No significant changes.	Post‐test comparisons between groups: Differences in TKA between PCEG and CG in favour of PCEG, SBTEG and CG in favour of SBTEG and PCEG and SBTEG in favour of SBTEG. Significant differences in LLA between PCEG and CG in favour of PCEG and SBTEG and CG in favour of SBTEG. In post‐test comparisons between groups: At equilibrium, difference between PCEG and CG in favour of PCEG.
Seidi et al. 2014	TKA. CG: reduction of only 0.64°. In LCEP and CCEP decrease of 5.04° and 12.25° respectively. Height changes: There was a significant difference between height levels before and after participation in the CCEP group.	Changes in forward head/shoulder angle. Significant difference in TKA change between LCEP and CCEP groups in favour of CCEP; LCEP and CG in favour of LECP and CCEP and CG in favour of CCEP.
Toprak Çelenay and Özer Kaya 2017	EG: Significant differences for postural back pain, central resistance, thoracic and lumbar curvature and general stability index. CG: No significant changes.	All parameters significantly different in EG compared to CG.
Yazici and Mohammadi 2017	EG: Significant difference in thoracic and lumbar curvature. CG: No significant change.	No significant inter‐group differences at each measurement point.

Abbreviations: A‐SLR, active straight leg raise; CCEP, comprehensive corrective exercise programme; CG, control group; EG, experimental group; INA, incline angle; LCEP, local corrective exercise programme; LLA, lumbar lordosis angle; PCEG, postural corrective exercise group; PPT, postural perception training; P‐SLR, passive straight leg raise; QoL, quality of life; ROM, rango de movimiento; SA, sacral angle; SBTEG, Schroth‐based three‐dimensional exercise group; SPBI, self‐perceived body image; SRS‐22, Scoliosis Research Society‐22 Questionnaire; TEP, thoracic exercise program; TKA, thoracic kyphosis angle.

## 4 Discussion

Currently, there is little evidence of the benefits of exercise for the reduction of hyperkyphosis in patients of different ages (Jenkins et al. 2021). Hence, the importance of this systematic review is to analyse the scientific evidence on the effect of therapeutic exercise in the treatment of hyperkyphosis in adolescents and young adults. As strengths, we find that therapeutic exercise is beneficial for both the TKA and other variables including the lumbar lordosis angle and balance.

With regard to methodological quality, four studies (Elpeze and Usgu 2022; Özdemir Görgü and Algun 2022; Seidi et al. 2014; Yazici and Mohammadi 2017) were of fair quality and five studies (Bezalel et al. 2019; Feng et al. 2018; Gheitasi et al. 2023; González‐Gálvez et al. 2022; Toprak Çelenay and Özer Kaya 2017) were of good quality. Among the criteria met by all the studies, criteria 10 (existence of intergroup statistical comparison) and 11 (indication of point measures and variability) are noteworthy. There were articles that could not score because there was no clear explanation of the inclusion criteria used (Seidi et al. 2014; Yazici and Mohammadi 2017), allocation to groups was not concealed (Elpeze and Usgu 2022; Feng et al. 2018; González‐Gálvez et al. 2022; Yazici and Mohammadi 2017), or there are baseline differences between groups (Bezalel et al. 2019). Furthermore, in some studies, there were losses of participants during follow‐up (Elpeze and Usgu 2022; Özdemir Görgü and Algun 2022; Seidi et al. 2014; Toprak Çelenay and Özer Kaya 2017; Yazici and Mohammadi 2017) thus measurements were not obtained from all subjects initially assigned to the groups.

In the same vein, the risk of bias was assessed according to the Cochrane handbook, with the majority of studies (Elpeze and Usgu 2022; Feng et al. 2018; Gheitasi et al. 2023; Özdemir Görgü and Algun 2022; Seidi et al. 2014; Toprak Çelenay and Özer Kaya 2017; Yazici and Mohammadi 2017) showing an unclear risk of implementation and detection bias. Notably, the results are consistent with studies that fail the following PEDro scale criteria: third (allocation was concealed) (Elpeze and Usgu 2022; Feng et al. 2018; González‐Gálvez et al. 2022; Seidi et al. 2014; Yazici and Mohammadi 2017), fifth (all subjects were blinded) (Elpeze and Usgu 2022; Feng et al. 2018; Gheitasi et al. 2023; González‐Gálvez et al. 2022; Özdemir Görgü and Algun 2022; Seidi et al. 2014), sixth (all therapists who administered therapy were blinded) (Bezalel et al. 2019; Elpeze and Usgu 2022; Feng et al. 2018; Gheitasi et al. 2023; González‐Gálvez et al. 2022; Özdemir Görgü and Algun 2022; Seidi et al. 2014; Toprak Çelenay and Özer Kaya 2017; Yazici and Mohammadi 2017) and seventh (all assessors who measured at least one key outcome were blinded) (Elpeze and Usgu 2022; Feng et al. 2018; Özdemir Görgü and Algun 2022; Seidi et al. 2014; Yazici and Mohammadi 2017).

The sample size varies between studies. Most of them included between 40 and 63 participants (Bezalel et al. 2019; Elpeze and Usgu 2022; Özdemir Görgü and Algun 2022; Seidi et al. 2014; Toprak Çelenay and Özer Kaya 2017; Yazici and Mohammadi 2017), with the exception of the studies by González‐Gálvez et al. (2022), Feng et al. (2018) and Gheitasi et al. (2023) with 97, 164 and 170 subjects, respectively. The estimate for the calculation of the sample size is reflected, except in Yazici and Mohammadi (2017) and Seidi et al. (2014). Regarding the characteristics of the subjects, most of the studies were carried out with both females and males, except in the studies by Yazici and Mohammadi (2017) and Elpeze and Usgu (2022), in which only men participated. This aspect should be considered because the prevalence of hyperkyphosis or hyperlordosis in females or males could influence the results. Some studies have reported a sex difference in kyphosis and lordosis angle, showing a higher TKA in men and a higher LLA in females (Bergenudd et al. 1989; Lang‐Tapia et al. 2011). However, others have found differences only in kyphosis (Endo et al. 2016) or isolated differences in lordosis (Lang‐Tapia et al. 2011). One study analysed sex differences in exercise effect on kyphosis angle, finding similar results between both (Katzman, Parimi, et al. 2017).

Hyperkyphosis was an inclusion criterion in all but three studies (Bezalel et al. 2019; Toprak Çelenay and Özer Kaya 2017; Yazici and Mohammadi 2017), defining it as a thoracic angle greater than 40° (Feng et al. 2018; González‐Gálvez et al. 2022; Özdemir Görgü and Algun 2022), 42° (Seidi et al. 2014), 45° (Gheitasi et al. 2023) and 50° (Elpeze and Usgu 2022). In Seidi et al. (2014), participants simultaneously presented excessive forward head (−44°) and forward shoulders (−49°), which are often associated with hyperkyphosis (Singla and Veqar 2017). Furthermore, it should be noted that the study by Bezalel et al. (2019) is the only study with adolescents diagnosed with Scheuermann's disease.

The nine studies analysed measured the TKA. Most of them used the Spinal Mouse system (Feng et al. 2018; González‐Gálvez et al. 2022; Özdemir Görgü and Algun 2022; Toprak Çelenay and Özer Kaya 2017; Yazici and Mohammadi 2017). However, in the studies by Elpeze and Usgu (2022) and Seidi et al. (2014), the Flexicurve method was used and in Gheitasi et al. (2023) and Bezalel et al. (2019), the radiography and Cobb method was used. Although there is evidence on the validity and reliability of the Spinal Mouse (Kellis et al. 2008; Livanelioglu et al. 2016; Mannion et al. 2004; Ripani et al. 2008) and flexicurve (de Oliveira et al. 2012; Lundon et al. 1998) for measuring spinal curvature, it should be considered that X‐rays provide greater accuracy. However, radiography requires X‐ray radiation (Harrison et al. 2001), has limited portability (Briggs et al. 2007; Teixeira and Carvalho 2007) and is more expensive (de Oliveira et al. 2012; Willner 1981).

Among the studies reviewed, there was variability not only in the content of the sessions but also in the duration of the programmes and their weekly frequency. However, in all nine studies (Bezalel et al. 2019; Elpeze and Usgu 2022; Feng et al. 2018; Gheitasi et al. 2023; González‐Gálvez et al. 2022; Özdemir Görgü and Algun 2022; Seidi et al. 2014; Toprak Çelenay and Özer Kaya 2017; Yazici and Mohammadi 2017), a significantly greater improvement in TKA was obtained in the experimental groups, in contrast to the controls. Six studies (Elpeze and Usgu 2022; Gheitasi et al. 2023; Özdemir Görgü and Algun 2022; Seidi et al. 2014; Toprak Çelenay and Özer Kaya 2017; Yazici and Mohammadi 2017) applied therapeutic exercise only to individuals in the experimental groups, with three of them (Elpeze and Usgu 2022; Özdemir Görgü and Algun 2022; Seidi et al. 2014) having two intervention groups receiving different exercise programmes and a control group with no exercise. The studies by González‐Gálvez et al. (2022), Feng et al. (2018) and Bezalel et al. (2019) compared two different exercise therapies. All investigations (Bezalel et al. 2019; Elpeze and Usgu 2022; Feng et al. 2018; Gheitasi et al. 2023; González‐Gálvez et al. 2022; Özdemir Görgü and Algun 2022; Seidi et al. 2014; Toprak Çelenay and Özer Kaya 2017; Yazici and Mohammadi 2017) used stretching and strengthening exercises in their intervention programme.

In two studies (Bezalel et al. 2019; Özdemir Görgü and Algun 2022), a group was treated with three‐dimensional exercise based on Schroth's method. A special feature of the study by Bezalel et al. (2019) is that the participants were adolescents diagnosed with Scheuermann's disease, in contrast to the study by Özdemir and Algun (8), in which the participants had postural kyphosis. In both investigations, the Schroth therapy group showed a significantly greater improvement in TKA. In the study by Bezalel et al. (2019), Schroth therapy was compared with anti‐gravity exercises and in the study by Özdemir Görgü and Algun (2022) it was compared a postural corrective exercise group and a group receiving only information. This greater improvement may be because the Schroth method is more effective for postural control. A recent review found that the Schroth method had a larger effect size in improving the Cobb angle than trunk stabilisation exercises (Dimitrijević et al. 2022).

Regarding how the sessions were conducted, Bezalel et al. (2019), Elpeze and Usgu (2022) and Seidi et al. (2014) specify that the interventions were carried out individually and Toprak Çelenay and Özer Kaya (2017), in groups. Furthermore, most studies specified exercise supervision (Bezalel et al. 2019; Elpeze and Usgu 2022; Gheitasi et al. 2023; Özdemir Görgü and Algun 2022; Seidi et al. 2014). However, although not detailed in the remaining four studies (Feng et al. 2018; González‐Gálvez et al. 2022; Toprak Çelenay and Özer Kaya 2017; Yazici and Mohammadi 2017), there does appear to be a displacement of participants towards centres and institutes. Therefore, with this coupled with the fact that TKA was the only variable assessed by all studies, it is difficult to conclude whether or not exercise supervision affects the results. It is also worth mentioning that Bezalel et al. (2019) asked for home exercises once a day and Elpeze and Usgu (2022) asked them to be aware of how often they notice and correct their bad posture in their daily life. Thus, with the information available, there seems to be a positive effect of exercise on TKA in adolescents and young adults, at least in the intervention variants observed among the studies reviewed (Bezalel et al. 2019; Elpeze and Usgu 2022; Feng et al. 2018; Gheitasi et al. 2023; González‐Gálvez et al. 2022; Özdemir Görgü and Algun 2022; Seidi et al. 2014; Toprak Çelenay and Özer Kaya 2017; Yazici and Mohammadi 2017). These findings are in agreement with previous reports in older adults (Bansal et al. 2014; González‐Gálvez et al. 2019).

On the other hand, five of the nine included studies (Feng et al. 2018; González‐Gálvez et al. 2022; Özdemir Görgü and Algun 2022; Toprak Çelenay and Özer Kaya 2017; Yazici and Mohammadi 2017) evaluate the LLA, finding a significant decrease, except in the study by Feng et al. (2018). However, it should be noted that Yazici and Mohammadi (2017) were the only studies in which the participants had hyperlordosis, that is LLA greater than 40° (González‐Gálvez et al. 2019). It should be noted that, compared to the other studies, Feng et al. (2018) had the lowest pre‐intervention LLA values and the shortest overall treatment time. Although it coincides with three studies (Özdemir Görgü and Algun 2022; Toprak Çelenay and Özer Kaya 2017; Yazici and Mohammadi 2017) in the duration of the exercise programme (8 weeks), its frequency and duration of sessions were lower. Regarding the intervention protocol, Feng et al. (2018) focused on improving range of motion, muscle strength and proprioception, including as such only one pectoral stretching exercise. Hyperlordosis is related to a shortening of the psoas iliacus and hamstrings and a lack of abdominal and paravertebral strengthening. Scientific evidence concludes that both stretching and strengthening are relevant in the management of lumbar lordosis (González‐Gálvez et al. 2019). Therefore, the lack of stretching exercises may have influenced less improvement in LLA in the study by Feng et al. (2018), although the initial non‐existence of hyperlordosis also contributes.

In terms of additional variables assessed, balance should be highlighted. In the current literature, most studies examining the effects of exercise to improve balance in people with thoracic hyperkyphosis involve older people, who are at an increased risk of falls (Bansal et al. 2014; Elpeze and Usgu 2022; Katzman et al. 2015, 2016; Katzman, Parimi, et al. 2017; Katzman et al. 2021; McDaniels‐Davidson et al. 2018; Tominaga et al. 2016). There is little research involving adolescents and young adults with hyperkyphosis (Jenkins et al. 2021). Three (Elpeze and Usgu 2022; Özdemir Görgü and Algun 2022; Toprak Çelenay and Özer Kaya 2017) of the nine included studies evaluated the effects of different exercise protocols on balance. For Elpeze and Usgu (2022), the group with a comprehensive corrective exercise programme is the only group with improvement of the Romberg index within the group, unlike the thoracic exercise programme and the control group. In the study by Özdemir Görgü and Algun (2022), a greater improvement in static postural stability balance scores was observed in the three‐dimensional Schroth‐based exercise group compared with the postural corrective exercise group. In Toprak Çelenay and Özer Kaya (2017), significant improvement was found only in the experimental group, based on teaching conscious and unconscious motor control, in contrast to the control group without exercise. The experimental groups with the greatest improvement had in common a training in postural perception and proprioception. However, the thoracic exercise programme and postural corrective exercise are focused on mobilisation, stretching and strengthening, which coincide with the therapies most commonly used by physiotherapists for the treatment of hyperkyphosis (Perriman et al. 2012).

As other aspects evaluated, it is worth mentioning that spinal deformities negatively affect pain and quality of life. Three studies included in this systematic review measure both variables (Bezalel et al. 2019; Özdemir Görgü and Algun 2022; Toprak Çelenay and Özer Kaya 2017), with improvement in at least one of the exercise protocols in all investigations. The improvement in pain and quality of life with exercise corresponds to previous reviews. In Gámiz‐Bermúdez et al. (2022), low quality evidence is shown for a large effect of corrective exercise‐based therapy intervention on the total measurement of the Scoliosis Research Society‐22 Questionnaire in adolescents with idiopathic scoliosis. In addition, the study by Ponzano et al. (2021) also suggests small improvements in quality of life and overall pain reduction following exercise in older people with hyperkyphosis. However, as in this review, none of the studies included were designed to measure quality of life or pain as a primary outcome. Therefore, further research into the effect of exercise on the quality of life and pain in people with spinal disorders is currently required.

Other variables studied, such as hamstring extensibility (González‐Gálvez et al. 2022), core strength (Toprak Çelenay and Özer Kaya 2017), height changes (Seidi et al. 2014) and forward head and shoulder angles (Seidi et al. 2014), were evaluated and significant improvements were obtained with the exercise protocols.

There are several limitations to reviewing whether therapeutic exercise is beneficial in the treatment of adolescents and young adults with hyperkyphosis. Currently, the protocols used in studies are too varied. Although it may be important to measure the effect of different protocols on TKA, there are other aspects such as functionality, range of motion, strength, or quality of life that seem not to have been taken into important consideration in most of the studies reviewed. As for the strengths of this review, it is worth highlighting that all the articles included are randomised clinical trials. In addition, it provides the different hyperkyphosis assessment tools, as well as the different detailed exercise protocols, which is interesting.

## 5 Implications on Physiotherapy Practice

The findings of this systematic review suggest that therapeutic exercise appears to be effective in the management of hyperkyphosis in adolescents and young adults. A positive effect of exercise on hyperkyphosis angle is sustained, at least in the intervention variants observed among the studies reviewed. In addition, benefits can be seen in other aspects such as LLA, balance, quality of life and pain. However, these variables are not subject to primary evaluation and the quality of the evidence found is fair to good. Therefore, the results of these studies should be interpreted with caution and indicate the need for further research.

The implication of the manuscript on the practice of physiotherapy is the contribution of knowledge on the efficacy of the application of therapeutic exercise as a form of treatment to be applied to patients with sagittal plane spinal involvement such as hyperkyphosis, which is commonly encountered in physiotherapy consultations.

In future lines of research, importance should be given to other aspects of hyperkyphosis in addition to TKA, such as more subjective aspects that affect quality of life such as pain, balance, or risk of falling, and to assess whether there are differences between males and females. Further follow‐up of the participants could also be done to check whether the effects are actually maintained over time.

## Ethics Statement

The authors have nothing to report.

## Consent

The authors have nothing to report.

## Conflicts of Interest

The authors declare no conflicts of interest.

## Permission to Reproduce Material From Other Sources

The authors have nothing to report.

## Data Availability

The authors have nothing to report.
